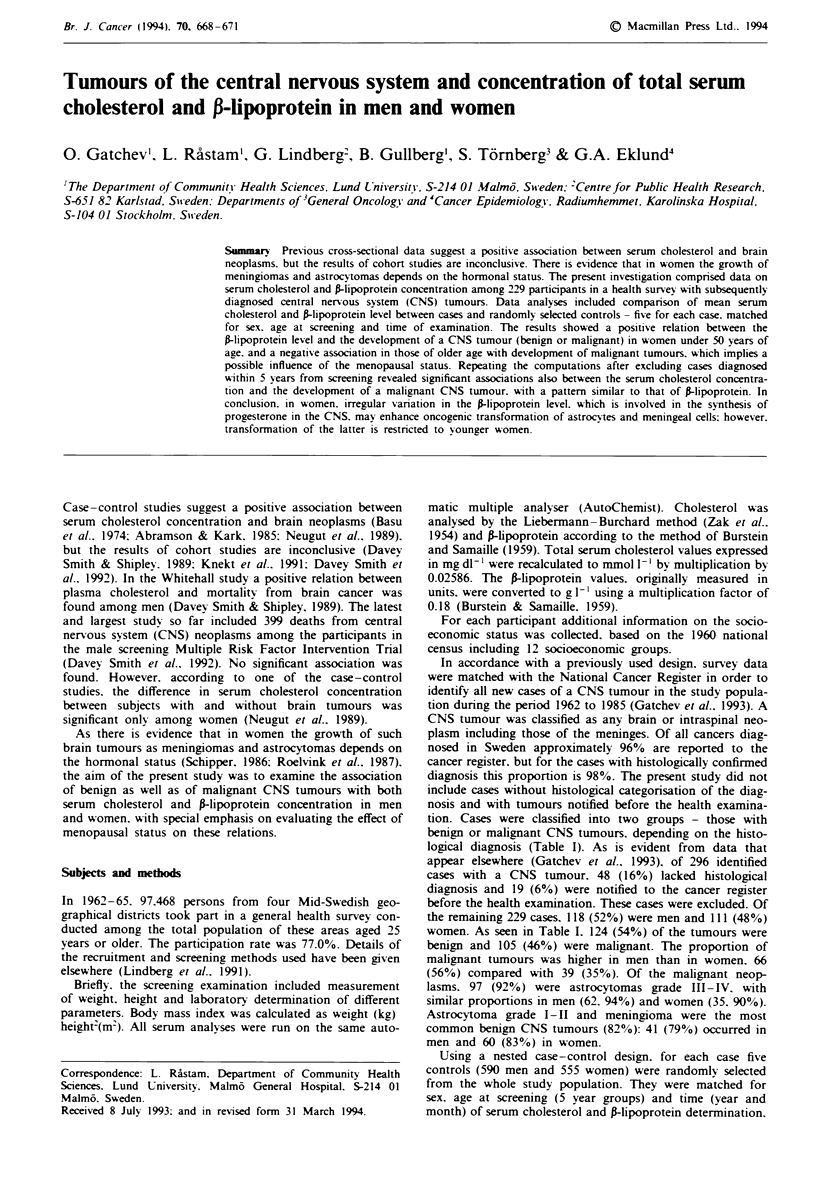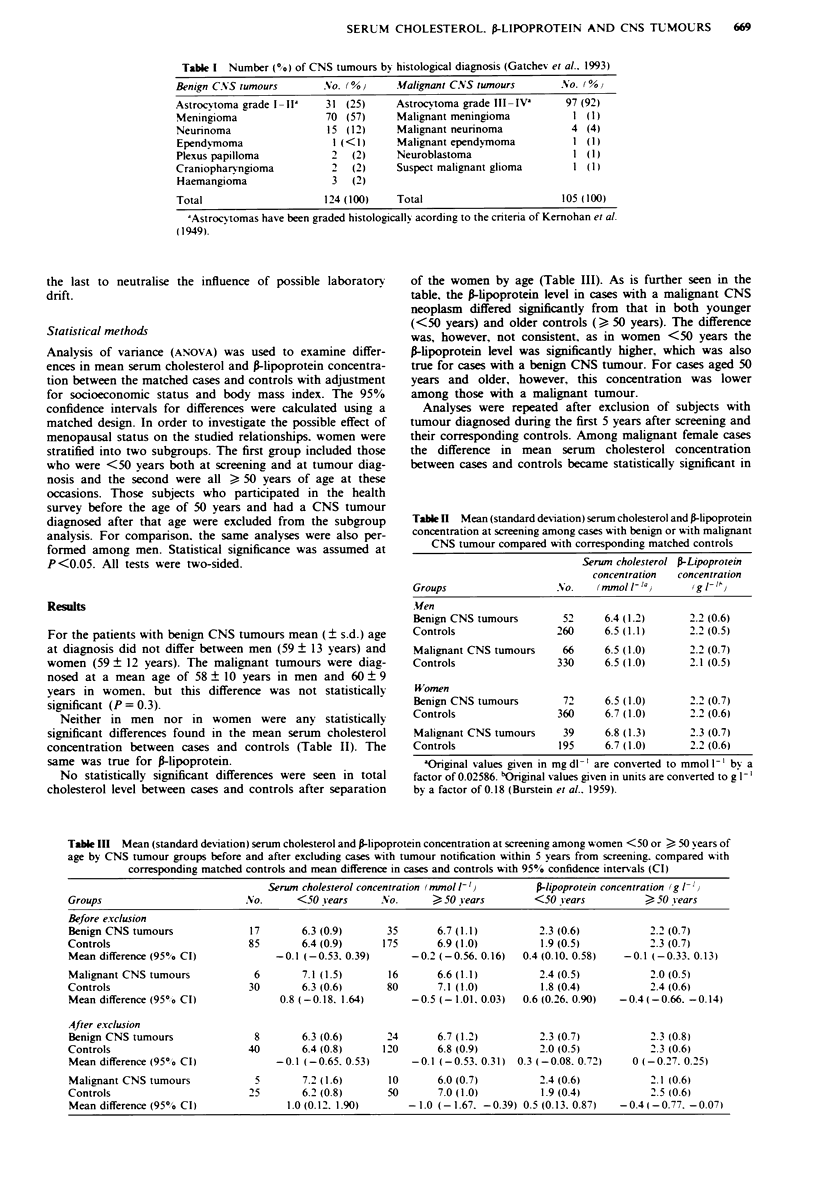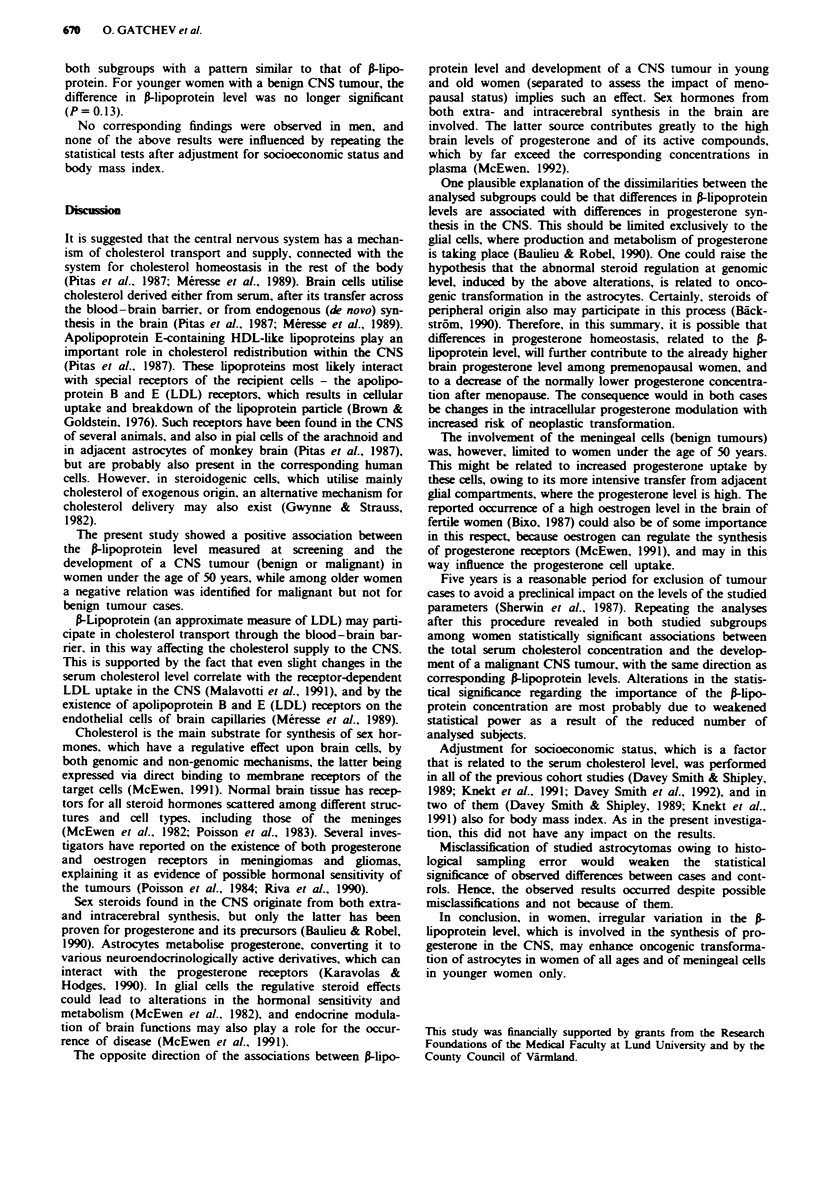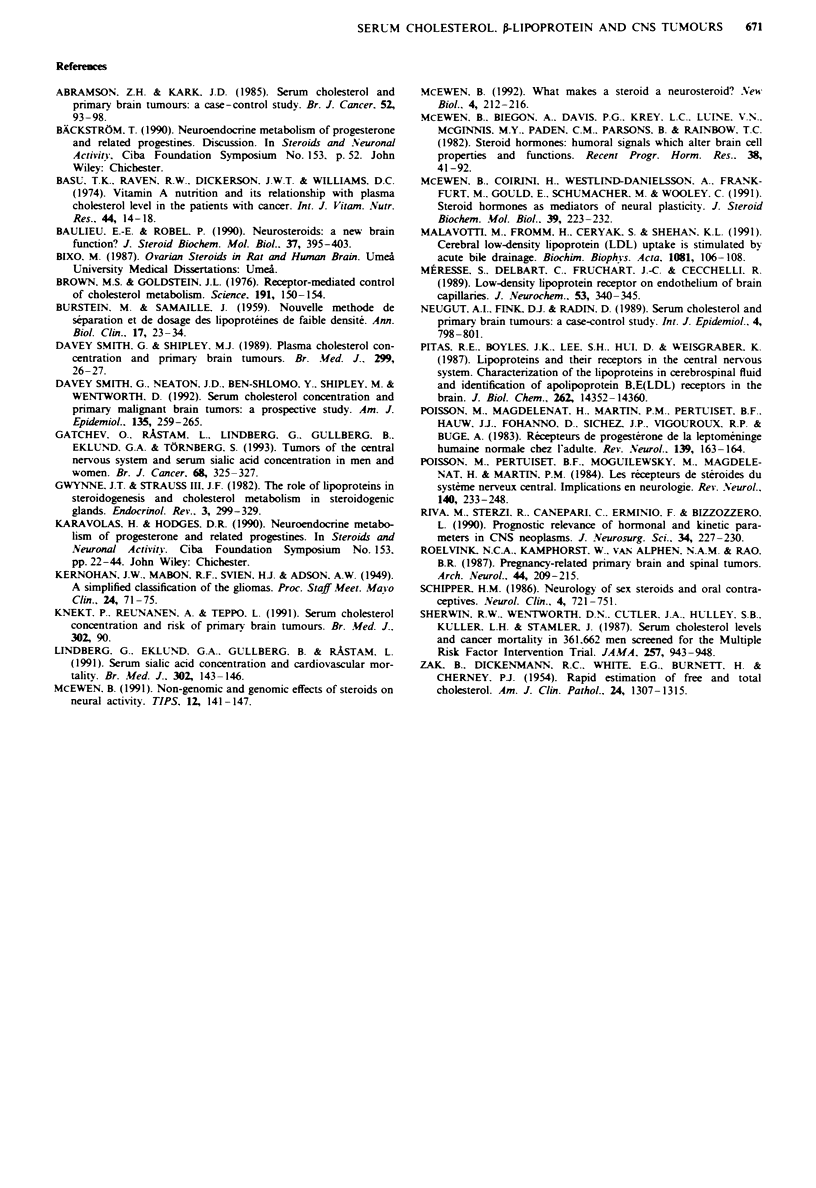# Tumours of the central nervous system and concentration of total serum cholesterol and beta-lipoprotein in men and women.

**DOI:** 10.1038/bjc.1994.368

**Published:** 1994-10

**Authors:** O. Gatchev, L. Råstam, G. Lindberg, B. Gullberg, S. Törnberg, G. A. Eklund

**Affiliations:** Department of Community Health Sciences, Lund University, Malmö, Sweden.

## Abstract

Previous cross-sectional data suggest a positive association between serum cholesterol and brain neoplasms, but the results of cohort studies are inconclusive. There is evidence that in women the growth of meningiomas and astrocytomas depends on the hormonal status. The present investigation comprised data on serum cholesterol and beta-lipoprotein concentration among 229 participants in a health survey with subsequently diagnosed central nervous system (CNS) tumours. Data analyses included comparison of mean serum cholesterol and beta-lipoprotein level between cases and randomly selected controls--five for each case, matched for sex, age at screening and time of examination. The results showed a positive relation between the beta-lipoprotein level and the development of a CNS tumour (benign or malignant) in women under 50 years of age, and a negative association in those of older age with development of malignant tumours, which implies a possible influence of the menopausal status. Repeating the computations after excluding cases diagnosed within 5 years from screening revealed significant associations also between the serum cholesterol concentration and the development of a malignant CNS tumour, with a pattern similar to that of beta-lipoprotein. In conclusion, in women, irregular variation in the beta-lipoprotein level, which is involved in the synthesis of progesterone in the CNS, may enhance oncogenic transformation of astrocytes and meningeal cells; however, transformation of the latter is restricted to younger women.


Br. J. Cancer (1994), 70, 668–671 © Macmillan Press Ltd., 1994

### Tumours of the central nervous system and concentration of total serum cholesterol and β-lipoprotein in men and women

O. Gatchev[1], L. Råstam[1], G. Lindberg[2], B. Gullberg[1], S. Törnberg[3] & G.A. Eklund[4]

[1]*The Department of Community Health Sciences, Lund University, S-214 01 Malmö, Sweden;* [2]*Centre for Public Health Research, S-651 82 Karlstad, Sweden; Departments of* [3]*General Oncology and* [4]*Cancer Epidemiology, Radiumhemmet, Karolinska Hospital, S-104 01 Stockholm, Sweden.*

**Summary** Previous cross-sectional data suggest a positive association between serum cholesterol and brain neoplasms, but the results of cohort studies are inconclusive. There is evidence that in women the growth of meningiomas and astrocytomas depends on the hormonal status. The present investigation comprised data on serum cholesterol and β-lipoprotein concentration among 229 participants in a health survey with subsequently diagnosed central nervous system (CNS) tumours. Data analyses included comparison of mean serum cholesterol and β-lipoprotein level between cases and randomly selected controls – five for each case, matched for sex, age at screening and time of examination. The results showed a positive relation between the β-lipoprotein level and the development of a CNS tumour (benign or malignant) in women under 50 years of age, and a negative association in those of older age with development of malignant tumours, which implies a possible influence of the menopausal status. Repeating the computations after excluding cases diagnosed within 5 years from screening revealed significant associations also between the serum cholesterol concentration and the development of a malignant CNS tumour, with a pattern similar to that of β-lipoprotein. In conclusion, in women, irregular variation in the β-lipoprotein level, which is involved in the synthesis of progesterone in the CNS, may enhance oncogenic transformation of astrocytes and meningeal cells; however, transformation of the latter is restricted to younger women.

Case–control studies suggest a positive association between serum cholesterol concentration and brain neoplasms (Basu *et al.*, 1974; Abramson & Kark, 1985; Neugut *et al.*, 1989), but the results of cohort studies are inconclusive (Davey Smith & Shipley, 1989; Knekt *et al.*, 1991; Davey Smith *et al.*, 1992). In the Whitehall study a positive relation between plasma cholesterol and mortality from brain cancer was found among men (Davey Smith & Shipley, 1989). The latest and largest study so far included 399 deaths from central nervous system (CNS) neoplasms among the participants in the male screening Multiple Risk Factor Intervention Trial (Davey Smith *et al.*, 1992). No significant association was found. However, according to one of the case–control studies, the difference in serum cholesterol concentration between subjects with and without brain tumours was significant only among women (Neugut *et al.*, 1989).

As there is evidence that in women the growth of such brain tumours as meningiomas and astrocytomas depends on the hormonal status (Schipper, 1986; Roelvink *et al.*, 1987), the aim of the present study was to examine the association of benign as well as of malignant CNS tumours with both serum cholesterol and β-lipoprotein concentration in men and women, with special emphasis on evaluating the effect of menopausal status on these relations.

### Subjects and methods

In 1962–65, 97,468 persons from four Mid-Swedish geographical districts took part in a general health survey conducted among the total population of these areas aged 25 years or older. The participation rate was 77.0%. Details of the recruitment and screening methods used have been given elsewhere (Lindberg *et al.*, 1991).

Briefly, the screening examination included measurement of weight, height and laboratory determination of different parameters. Body mass index was calculated as weight (kg) height$^2$(m$^2$). All serum analyses were run on the same automatic multiple analyser (AutoChemist). Cholesterol was analysed by the Liebermann–Burchard method (Zak *et al.*, 1954) and β-lipoprotein according to the method of Burstein and Samaille (1959). Total serum cholesterol values expressed in mg dl$^{-1}$ were recalculated to mmol l$^{-1}$ by multiplication by 0.02586. The β-lipoprotein values, originally measured in units, were converted to g l$^{-1}$ using a multiplication factor of 0.18 (Burstein & Samaille, 1959).

For each participant additional information on the socioeconomic status was collected, based on the 1960 national census including 12 socioeconomic groups.

In accordance with a previously used design, survey data were matched with the National Cancer Register in order to identify all new cases of a CNS tumour in the study population during the period 1962 to 1985 (Gatchev *et al.*, 1993). A CNS tumour was classified as any brain or intraspinal neoplasm including those of the meninges. Of all cancers diagnosed in Sweden approximately 96% are reported to the cancer register, but for the cases with histologically confirmed diagnosis this proportion is 98%. The present study did not include cases without histological categorisation of the diagnosis and with tumours notified before the health examination. Cases were classified into two groups – those with benign or malignant CNS tumours, depending on the histological diagnosis (Table I). As is evident from data that appear elsewhere (Gatchev *et al.*, 1993), of 296 identified cases with a CNS tumour, 48 (16%) lacked histological diagnosis and 19 (6%) were notified to the cancer register before the health examination. These cases were excluded. Of the remaining 229 cases, 118 (52%) were men and 111 (48%) women. As seen in Table I, 124 (54%) of the tumours were benign and 105 (46%) were malignant. The proportion of malignant tumours was higher in men than in women, 66 (56%) compared with 39 (35%). Of the malignant neoplasms, 97 (92%) were astrocytomas grade III–IV, with similar proportions in men (62, 94%) and women (35, 90%). Astrocytoma grade I–II and meningioma were the most common benign CNS tumours (82%): 41 (79%) occurred in men and 60 (83%) in women.

Using a nested case–control design, for each case five controls (590 men and 555 women) were randomly selected from the whole study population. They were matched for sex, age at screening (5 year groups) and time (year and month) of serum cholesterol and β-lipoprotein determination.

Correspondence: L. Råstam, Department of Community Health Sciences, Lund University, Malmö General Hospital, S-214 01 Malmö, Sweden.

Received 8 July 1993; and in revised form 31 March 1994.

**Table I** Number (%) of CNS tumours by histological diagnosis (Gatchev *et al.*, 1993)

| *Benign CNS tumours* | *No. (%)* | *Malignant CNS tumours* | *No. (%)* |
|---|---|---|---|
| Astrocytoma grade I-II[a] | 31 (25) | Astrocytoma grade III-IV[a] | 97 (92) |
| Meningioma | 70 (57) | Malignant meningioma | 1 (1) |
| Neurinoma | 15 (12) | Malignant neurinoma | 4 (4) |
| Ependymoma | 1 (<1) | Malignant ependymoma | 1 (1) |
| Plexus papilloma | 2 (2) | Neuroblastoma | 1 (1) |
| Craniopharyngioma | 2 (2) | Suspect malignant glioma | 1 (1) |
| Haemangioma | 3 (2) | | |
| Total | 124 (100) | Total | 105 (100) |

[a]Astrocytomas have been graded histologically acording to the criteria of Kernohan *et al.* (1949).

the last to neutralise the influence of possible laboratory drift.

*Statistical methods*

Analysis of variance (ANOVA) was used to examine differences in mean serum cholesterol and β-lipoprotein concentration between the matched cases and controls with adjustment for socioeconomic status and body mass index. The 95% confidence intervals for differences were calculated using a matched design. In order to investigate the possible effect of menopausal status on the studied relationships, women were stratified into two subgroups. The first group included those who were <50 years both at screening and at tumour diagnosis and the second were all ⩾ 50 years of age at these occasions. Those subjects who participated in the health survey before the age of 50 years and had a CNS tumour diagnosed after that age were excluded from the subgroup analysis. For comparison, the same analyses were also performed among men. Statistical significance was assumed at $P<0.05$. All tests were two-sided.

### Results

For the patients with benign CNS tumours mean (± s.d.) age at diagnosis did not differ between men (59 ± 13 years) and women (59 ± 12 years). The malignant tumours were diagnosed at a mean age of 58 ± 10 years in men and 60 ± 9 years in women, but this difference was not statistically significant ($P = 0.3$).

Neither in men nor in women were any statistically significant differences found in the mean serum cholesterol concentration between cases and controls (Table II). The same was true for β-lipoprotein.

No statistically significant differences were seen in total cholesterol level between cases and controls after separation of the women by age (Table III). As is further seen in the table, the β-lipoprotein level in cases with a malignant CNS neoplasm differed significantly from that in both younger (<50 years) and older controls (⩾ 50 years). The difference was, however, not consistent, as in women <50 years the β-lipoprotein level was significantly higher, which was also true for cases with a benign CNS tumour. For cases aged 50 years and older, however, this concentration was lower among those with a malignant tumour.

Analyses were repeated after exclusion of subjects with tumour diagnosed during the first 5 years after screening and their corresponding controls. Among malignant female cases the difference in mean serum cholesterol concentration between cases and controls became statistically significant in

**Table II** Mean (standard deviation) serum cholesterol and β-lipoprotein concentration at screening among cases with benign or with malignant CNS tumour compared with corresponding matched controls

| *Groups* | *No.* | *Serum cholesterol concentration (mmol l⁻¹ᵃ)* | *β-Lipoprotein concentration (g l⁻¹ᵇ)* |
|---|---|---|---|
| *Men* | | | |
| Benign CNS tumours | 52 | 6.4 (1.2) | 2.2 (0.6) |
| Controls | 260 | 6.5 (1.1) | 2.2 (0.5) |
| Malignant CNS tumours | 66 | 6.5 (1.0) | 2.2 (0.7) |
| Controls | 330 | 6.5 (1.0) | 2.1 (0.5) |
| *Women* | | | |
| Benign CNS tumours | 72 | 6.5 (1.0) | 2.2 (0.7) |
| Controls | 360 | 6.7 (1.0) | 2.2 (0.6) |
| Malignant CNS tumours | 39 | 6.8 (1.3) | 2.3 (0.7) |
| Controls | 195 | 6.7 (1.0) | 2.2 (0.6) |

[a]Original values given in mg dl$^{-1}$ are converted to mmol l$^{-1}$ by a factor of 0.02586. [b]Original values given in units are converted to g l$^{-1}$ by a factor of 0.18 (Burstein *et al.*, 1959).

**Table III** Mean (standard deviation) serum cholesterol and β-lipoprotein concentration at screening among women <50 or ⩾ 50 years of age by CNS tumour groups before and after excluding cases with tumour notification within 5 years from screening, compared with corresponding matched controls and mean difference in cases and controls with 95% confidence intervals (CI)

| *Groups* | *No.* | *Serum cholesterol concentration (mmol l⁻¹)* <50 years | *No.* | ⩾50 years | *β-lipoprotein concentration (g l⁻¹)* <50 years | ⩾50 years |
|---|---|---|---|---|---|---|
| *Before exclusion* | | | | | | |
| Benign CNS tumours | 17 | 6.3 (0.9) | 35 | 6.7 (1.1) | 2.3 (0.6) | 2.2 (0.7) |
| Controls | 85 | 6.4 (0.9) | 175 | 6.9 (1.0) | 1.9 (0.5) | 2.3 (0.7) |
| Mean difference (95% CI) | | −0.1 (−0.53, 0.39) | | −0.2 (−0.56, 0.16) | 0.4 (0.10, 0.58) | −0.1 (−0.33, 0.13) |
| Malignant CNS tumours | 6 | 7.1 (1.5) | 16 | 6.6 (1.1) | 2.4 (0.5) | 2.0 (0.5) |
| Controls | 30 | 6.3 (0.6) | 80 | 7.1 (1.0) | 1.8 (0.4) | 2.4 (0.6) |
| Mean difference (95% CI) | | 0.8 (−0.18, 1.64) | | −0.5 (−1.01, 0.03) | 0.6 (0.26, 0.90) | −0.4 (−0.66, −0.14) |
| *After exclusion* | | | | | | |
| Benign CNS tumours | 8 | 6.3 (0.6) | 24 | 6.7 (1.2) | 2.3 (0.7) | 2.3 (0.8) |
| Controls | 40 | 6.4 (0.8) | 120 | 6.8 (0.9) | 2.0 (0.5) | 2.3 (0.6) |
| Mean difference (95% CI) | | −0.1 (−0.65, 0.53) | | −0.1 (−0.53, 0.31) | 0.3 (−0.08, 0.72) | 0 (−0.27, 0.25) |
| Malignant CNS tumours | 5 | 7.2 (1.6) | 10 | 6.0 (0.7) | 2.4 (0.6) | 2.1 (0.6) |
| Controls | 25 | 6.2 (0.8) | 50 | 7.0 (1.0) | 1.9 (0.4) | 2.5 (0.6) |
| Mean difference (95% CI) | | 1.0 (0.12, 1.90) | | −1.0 (−1.67, −0.39) | 0.5 (0.13, 0.87) | −0.4 (−0.77, −0.07) |

both subgroups with a pattern similar to that of β-lipoprotein. For younger women with a benign CNS tumour, the difference in β-lipoprotein level was no longer significant ($P = 0.13$).

No corresponding findings were observed in men, and none of the above results were influenced by repeating the statistical tests after adjustment for socioeconomic status and body mass index.

### Discussion

It is suggested that the central nervous system has a mechanism of cholesterol transport and supply, connected with the system for cholesterol homeostasis in the rest of the body (Pitas et al., 1987; Méresse et al., 1989). Brain cells utilise cholesterol derived either from serum, after its transfer across the blood–brain barrier, or from endogenous (*de novo*) synthesis in the brain (Pitas et al., 1987; Méresse et al., 1989). Apolipoprotein E-containing HDL-like lipoproteins play an important role in cholesterol redistribution within the CNS (Pitas et al., 1987). These lipoproteins most likely interact with special receptors of the recipient cells – the apolipoprotein B and E (LDL) receptors, which results in cellular uptake and breakdown of the lipoprotein particle (Brown & Goldstein, 1976). Such receptors have been found in the CNS of several animals, and also in pial cells of the arachnoid and in adjacent astrocytes of monkey brain (Pitas et al., 1987), but are probably also present in the corresponding human cells. However, in steroidogenic cells, which utilise mainly cholesterol of exogenous origin, an alternative mechanism for cholesterol delivery may also exist (Gwynne & Strauss, 1982).

The present study showed a positive association between the β-lipoprotein level measured at screening and the development of a CNS tumour (benign or malignant) in women under the age of 50 years, while among older women a negative relation was identified for malignant but not for benign tumour cases.

β-Lipoprotein (an approximate measure of LDL) may participate in cholesterol transport through the blood–brain barrier, in this way affecting the cholesterol supply to the CNS. This is supported by the fact that even slight changes in the serum cholesterol level correlate with the receptor-dependent LDL uptake in the CNS (Malavotti et al., 1991), and by the existence of apolipoprotein B and E (LDL) receptors on the endothelial cells of brain capillaries (Méresse et al., 1989).

Cholesterol is the main substrate for synthesis of sex hormones, which have a regulative effect upon brain cells, by both genomic and non-genomic mechanisms, the latter being expressed via direct binding to membrane receptors of the target cells (McEwen, 1991). Normal brain tissue has receptors for all steroid hormones scattered among different structures and cell types, including those of the meninges (McEwen et al., 1982; Poisson et al., 1983). Several investigators have reported on the existence of both progesterone and oestrogen receptors in meningiomas and gliomas, explaining it as evidence of possible hormonal sensitivity of the tumours (Poisson et al., 1984; Riva et al., 1990).

Sex steroids found in the CNS originate from both extra- and intracerebral synthesis, but only the latter has been proven for progesterone and its precursors (Baulieu & Robel, 1990). Astrocytes metabolise progesterone, converting it to various neuroendocrinologically active derivatives, which can interact with the progesterone receptors (Karavolas & Hodges, 1990). In glial cells the regulative steroid effects could lead to alterations in the hormonal sensitivity and metabolism (McEwen et al., 1982), and endocrine modulation of brain functions may also play a role for the occurrence of disease (McEwen et al., 1991).

The opposite direction of the associations between β-lipoprotein level and development of a CNS tumour in young and old women (separated to assess the impact of menopausal status) implies such an effect. Sex hormones from both extra- and intracerebral synthesis in the brain are involved. The latter source contributes greatly to the high brain levels of progesterone and of its active compounds, which by far exceed the corresponding concentrations in plasma (McEwen, 1992).

One plausible explanation of the dissimilarities between the analysed subgroups could be that differences in β-lipoprotein levels are associated with differences in progesterone synthesis in the CNS. This should be limited exclusively to the glial cells, where production and metabolism of progesterone is taking place (Baulieu & Robel, 1990). One could raise the hypothesis that the abnormal steroid regulation at genomic level, induced by the above alterations, is related to oncogenic transformation in the astrocytes. Certainly, steroids of peripheral origin also may participate in this process (Bäckström, 1990). Therefore, in this summary, it is possible that differences in progesterone homeostasis, related to the β-lipoprotein level, will further contribute to the already higher brain progesterone level among premenopausal women, and to a decrease of the normally lower progesterone concentration after menopause. The consequence would in both cases be changes in the intracellular progesterone modulation with increased risk of neoplastic transformation.

The involvement of the meningeal cells (benign tumours) was, however, limited to women under the age of 50 years. This might be related to increased progesterone uptake by these cells, owing to its more intensive transfer from adjacent glial compartments, where the progesterone level is high. The reported occurrence of a high oestrogen level in the brain of fertile women (Bixo, 1987) could also be of some importance in this respect, because oestrogen can regulate the synthesis of progesterone receptors (McEwen, 1991), and may in this way influence the progesterone cell uptake.

Five years is a reasonable period for exclusion of tumour cases to avoid a preclinical impact on the levels of the studied parameters (Sherwin et al., 1987). Repeating the analyses after this procedure revealed in both studied subgroups among women statistically significant associations between the total serum cholesterol concentration and the development of a malignant CNS tumour, with the same direction as corresponding β-lipoprotein levels. Alterations in the statistical significance regarding the importance of the β-lipoprotein concentration are most probably due to weakened statistical power as a result of the reduced number of analysed subjects.

Adjustment for socioeconomic status, which is a factor that is related to the serum cholesterol level, was performed in all of the previous cohort studies (Davey Smith & Shipley, 1989; Knekt et al., 1991; Davey Smith et al., 1992), and in two of them (Davey Smith & Shipley, 1989; Knekt et al., 1991) also for body mass index. As in the present investigation, this did not have any impact on the results.

Misclassification of studied astrocytomas owing to histological sampling error would weaken the statistical significance of observed differences between cases and controls. Hence, the observed results occurred despite possible misclassifications and not because of them.

In conclusion, in women, irregular variation in the β-lipoprotein level, which is involved in the synthesis of progesterone in the CNS, may enhance oncogenic transformation of astrocytes in women of all ages and of meningeal cells in younger women only.

This study was financially supported by grants from the Research Foundations of the Medical Faculty at Lund University and by the County Council of Värmland.

**References**

ABRAMSON, Z.H. & KARK, J.D. (1985). Serum cholesterol and primary brain tumours: a case-control study. *Br. J. Cancer*, **52**, 93-98.

BÄCKSTRÖM, T. (1990). Neuroendocrine metabolism of progesterone and related progestines. Discussion. In *Steroids and Neuronal Activity*, Ciba Foundation Symposium No. 153, p. 52. John Wiley: Chichester.

BASU, T.K., RAVEN, R.W., DICKERSON, J.W.T. & WILLIAMS, D.C. (1974). Vitamin A nutrition and its relationship with plasma cholesterol level in the patients with cancer. *Int. J. Vitam. Nutr. Res.*, **44**, 14-18.

BAULIEU, E.-E. & ROBEL, P. (1990). Neurosteroids: a new brain function? *J. Steroid Biochem. Mol. Biol.*, **37**, 395-403.

BIXO, M. (1987). *Ovarian Steroids in Rat and Human Brain*. Umeå University Medical Dissertations: Umeå.

BROWN, M.S. & GOLDSTEIN, J.L. (1976). Receptor-mediated control of cholesterol metabolism. *Science*, **191**, 150-154.

BURSTEIN, M. & SAMAILLE, J. (1959). Nouvelle methode de séparation et de dosage des lipoprotéines de faible densité. *Ann. Biol. Clin.*, **17**, 23-34.

DAVEY SMITH, G. & SHIPLEY, M.J. (1989). Plasma cholesterol concentration and primary brain tumours. *Br. Med. J.*, **299**, 26-27.

DAVEY SMITH, G., NEATON, J.D., BEN-SHLOMO, Y., SHIPLEY, M. & WENTWORTH, D. (1992). Serum cholesterol concentration and primary malignant brain tumors: a prospective study. *Am. J. Epidemiol.*, **135**, 259-265.

GATCHEV, O., RÅSTAM, L., LINDBERG, G., GULLBERG, B., EKLUND, G.A. & TÖRNBERG, S. (1993). Tumors of the central nervous system and serum sialic acid concentration in men and women. *Br. J. Cancer*, **68**, 325-327.

GWYNNE, J.T. & STRAUSS III, J.F. (1982). The role of lipoproteins in steroidogenesis and cholesterol metabolism in steroidogenic glands. *Endocrinol. Rev.*, **3**, 299-329.

KARAVOLAS, H. & HODGES, D.R. (1990). Neuroendocrine metabolism of progesterone and related progestines. In *Steroids and Neuronal Activity*. Ciba Foundation Symposium No. 153, pp. 22-44. John Wiley: Chichester.

KERNOHAN, J.W., MABON, R.F., SVIEN, H.J. & ADSON, A.W. (1949). A simplified classification of the gliomas. *Proc. Staff Meet. Mayo Clin.*, **24**, 71-75.

KNEKT, P., REUNANEN, A. & TEPPO, L. (1991). Serum cholesterol concentration and risk of primary brain tumours. *Br. Med. J.*, **302**, 90.

LINDBERG, G., EKLUND, G.A., GULLBERG, B. & RÅSTAM, L. (1991). Serum sialic acid concentration and cardiovascular mortality. *Br. Med. J.*, **302**, 143-146.

McEWEN, B. (1991). Non-genomic and genomic effects of steroids on neural activity. *TIPS*, **12**, 141-147.

McEWEN, B. (1992). What makes a steroid a neurosteroid? *New Biol.*, **4**, 212-216.

McEWEN, B., BIEGON, A., DAVIS, P.G., KREY, L.C., LUINE, V.N., McGINNIS, M.Y., PADEN, C.M., PARSONS, B. & RAINBOW, T.C. (1982). Steroid hormones: humoral signals which alter brain cell properties and functions. *Recent Progr. Horm. Res.*, **38**, 41-92.

McEWEN, B., COIRINI, H., WESTLIND-DANIELSSON, A., FRANKFURT, M., GOULD, E., SCHUMACHER, M. & WOOLEY, C. (1991). Steroid hormones as mediators of neural plasticity. *J. Steroid Biochem. Mol. Biol.*, **39**, 223-232.

MALAVOTTI, M., FROMM, H., CERYAK, S. & SHEHAN, K.L. (1991). Cerebral low-density lipoprotein (LDL) uptake is stimulated by acute bile drainage. *Biochim. Biophys. Acta*, **1081**, 106-108.

MÉRESSE, S., DELBART, C., FRUCHART, J.-C. & CECCHELLI, R. (1989). Low-density lipoprotein receptor on endothelium of brain capillaries. *J. Neurochem.*, **53**, 340-345.

NEUGUT, A.I., FINK, D.J. & RADIN, D. (1989). Serum cholesterol and primary brain tumours: a case-control study. *Int. J. Epidemiol.*, **4**, 798-801.

PITAS, R.E., BOYLES, J.K., LEE, S.H., HUI, D. & WEISGRABER, K. (1987). Lipoproteins and their receptors in the central nervous system. Characterization of the lipoproteins in cerebrospinal fluid and identification of apolipoprotein B,E(LDL) receptors in the brain. *J. Biol. Chem.*, **262**, 14352-14360.

POISSON, M., MAGDELENAT, H., MARTIN, P.M., PERTUISET, B.F., HAUW, J.J., FOHANNO, D., SICHEZ, J.P., VIGOUROUX, R.P. & BUGE, A. (1983). Récepteurs de progestérone de la leptoméninge humaine normale chez l'adulte. *Rev. Neurol.*, **139**, 163-164.

POISSON, M., PERTUISET, B.F., MOGUILEWSKY, M., MAGDELENAT, H. & MARTIN, P.M. (1984). Les récepteurs de stéroides du système nerveux central. Implications en neurologie. *Rev. Neurol.*, **140**, 233-248.

RIVA, M., STERZI, R., CANEPARI, C., ERMINIO, F. & BIZZOZZERO, L. (1990). Prognostic relevance of hormonal and kinetic parameters in CNS neoplasms. *J. Neurosurg. Sci.*, **34**, 227-230.

ROELVINK, N.C.A., KAMPHORST, W., VAN ALPHEN, N.A.M. & RAO, B.R. (1987). Pregnancy-related primary brain and spinal tumors. *Arch. Neurol.*, **44**, 209-215.

SCHIPPER, H.M. (1986). Neurology of sex steroids and oral contraceptives. *Neurol. Clin.*, **4**, 721-751.

SHERWIN, R.W., WENTWORTH, D.N., CUTLER, J.A., HULLEY, S.B., KULLER, L.H. & STAMLER, J. (1987). Serum cholesterol levels and cancer mortality in 361,662 men screened for the Multiple Risk Factor Intervention Trial. *JAMA*, **257**, 943-948.

ZAK, B., DICKENMANN, R.C., WHITE, E.G., BURNETT, H. & CHERNEY, P.J. (1954). Rapid estimation of free and total cholesterol. *Am. J. Clin. Pathol.*, **24**, 1307-1315.